# Creating a Geography of Access for Rural Older Adult Mobility

**DOI:** 10.1093/geroni/igaf122.1290

**Published:** 2025-12-31

**Authors:** Cara Marcus

**Affiliations:** National Rural Transit Assistance Program, Woburn, Massachusetts, United States

## Abstract

Older adults in rural areas often lack access to transportation that is available in cities, suburbs, and large towns. Challenges to providing transportation in rural areas include lack of funding and resources, long distances, and difficult-to-navigate terrain. National Rural Transit Assistance Program (National RTAP) analyzed data on the breakdown of rural older adults asking for assistance in locating rides between 2016 and 2019 (n = 31). Findings indicated that 38% needed transportation for medical appointments, 23% needed general transportation, and the remainder was evenly distributed between transportation to grocery stores, assisted living facilities, hospitals, dialysis centers, and leisure destinations. We also found that rural communities are working towards creating more access for older adults through innovative transportation solutions, such as ride-sharing, transportation brokerages, feeder routes, nutrition shuttles, and more. Through collaboration with organizations like Area Agencies on Aging, community action councils, mobility managers, elected officials, and others, rural transit agencies can work to address the full spectrum of mobility needs of this important population. National RTAP has created guidance for all stakeholders on assisting people with finding transportation that includes situational assessment, research processes, databases and tools, and recommendations for relaying information to older adults.

